# Morbidity, Mortality, and Short-Term Outcomes of Preterm Infants ≤ 25 Weeks of Gestation

**DOI:** 10.3390/jcm15093198

**Published:** 2026-04-22

**Authors:** Melinda Matyas, Florica Ramona Dorobantu, Madalina Valeanu, Monica G. Hasmasanu, Nicoleta Grosu, Adelina Tutu, Anna D. Jakab, Gabriela Zaharie

**Affiliations:** 1 Neonatology Department, “Iuliu Hatieganu” University of Medicine and Pharmacy, 400012 Cluj-Napoca, Romania; 2 Department of Medical Discipline, Faculty of Medicine and Pharmacy, University of Oradea, 410087 Oradea, Romania; 3 Medical Informatics and Biostatistics Department, “Iuliu Hatieganu” University of Medicine and Pharmacy, 400012 Cluj-Napoca, Romania; 4 Neonatology Department, County Emergency Hospital, 400006 Cluj-Napoca, Romania; 5 Faculty of Medicine, “Iuliu Hatieganu” University of Medicine and Pharmacy, 400012 Cluj-Napoca, Romania

**Keywords:** extremely preterm, mortality rate, in-hospital morbidity, neurodevelopmental impairment

## Abstract

**Background:** Short-term morbidities and mortality decreased significantly in the past decade at preterm born < 25 weeks of gestation. Severe lifelong morbidities affect an important part of these patients. Objective: to investigate the in-hospital morbidity, mortality, and short-term complications of preterm neonates born ≤25 weeks of gestation. **Methods:** A prospective longitudinal cohort study was conducted in children born 2021–2024, ≤25 weeks of gestation, admitted to a 3rd-level unit, and care till discharge. Pregnancy complications’ effect on neonatal evolution was analyzed, six main in-hospital morbidities specific for preterm birth and other aggravating circumstances, with a possible effect on the evolution were analyzed, as follows: inflammatory syndrome, early pulmonary or digestive hemorrhages, and early inotropic support. The neurological development in the first year of life was analyzed through theparticipation of premature infants in the follow-up program after discharge. **Results:** Forty-nine premature infants were enrolled, with a mean gestational age of 24.37 ± 0.76 weeks and an average weight of 665 ± 143 g. Most newborns required intubation at birth (42/49), and 33/49 received 2-dose surfactant therapy postnatally. NEC was present in 26.5% of the group, being more common in patients with inflammatory syndrome—increase in procalcitonin (PCT), and those who received a higher number of blood transfusions. The BPD and ROP, as well as the severity of the latter, correlated with the oxygen requirement on the 28th day of life. BPD was more common in infants associated with PDA requiring combination treatment. ROP increased with the number of transfusions required by patients. At the follow-up at the first timepoint evaluation, were 51% of the study group, and 30.6% of them had normal neurological development. At 12 months of age, however, the neurological examination was normal in only three patients (23.08%) but only 36.5% of the study group attended the follow-up. Neurodevelopmental disorders were present in 10 of the patients, one with spastic diplegia. **Conclusions:** In the hospital, the morbidity and survival rate of the group was like other studies. The small number of follow-up participants does not allow the generalization of the data, but as far as neurological development is concerned, it is like that of other studies.

## 1. Introduction

In the past decade, with active treatment, the survival of infants born at <25 weeks’ gestation has increased. Recent decades have also seen a decline in most short-term morbidities, yet as care strategies and technologies continue to evolve, medical and neurodevelopmental outcomes will need ongoing review.

The Neonatal Research Network of Japan (NRNJ) reported a decrease in the combined incidence of death or neurodevelopmental impairments (NDI) in infants born before 25 weeks’ gestation, from 54% in 2003–2007 to 47% in 2008–2012 [1]. In 2003–2005, the same network reported that at a chronological age of 36–42 months, those born at 22 weeks had an incidence of death or NDI of 80%, and those born at 23 weeks had an incidence of 64% [1].

Bell et al. reported in their study of the population from the Eunice Kennedy Shriver National Institute of Child Health and Human Development Neonatal Research Network that only 21.2% had severe NDI, while 29.3% had moderate NDI, and 48.7% of the evaluated children had no or mild NDI [2].

Extreme preterm birth is associated with severe life-long morbidities, not just NDI, but also important intellectual impairment (an IQ < 55), and severe sensory problems like blindness or hypoacusis [3,4,5,6].

Variation in outcomes is likely influenced by a combination of prenatal characteristics and postnatal factors, such as care, interventions, and morbidities in neonatal intensive care units.

Stoll et al. reported a significant increase in survival rates in deliveries at 23-, 24-, and 25 weeks’ gestation and no improvement at 22 weeks’ [5]. Further studies reported that morbidity-free discharge rates showed improvement at 25 weeks’ gestation and beyond, with no significant improvement in infants born between 22 and 24 weeks [7,8,9,10]. These findings align with evidence suggesting that perinatal mortality among extremely preterm infants across all gestational ages is strongly shaped by clinical decision-making regarding the provision of active interventions and by delivery in higher-care settings [11,12,13]. The EPICure study 2 reports improved survival for infants born at 22–26 weeks in a level 3 unit, which supports the full range of neonatal intensive care services, but no improvements in neonatal morbidity [11]. Whilst the capacity to sustain extremely preterm infants has advanced, a ceiling effect in morbidity-free survival at the lowest gestational ages appears to persist and is explained by either antenatal injury or the limitations of current life-support technologies [5,9,14]. Considering the complexity of cases in premature babies born at gestational age less than 26 weeks, it is recommended to apply individualized care, adapted to each case [5,15]. Extremely pre-term infants carry a heightened risk of both acute and long-term morbidities stemming from the underlying causes of preterm delivery, early exposure to the extra-uterine environment, and iatrogenic effects associated with life-support interventions [7,11,12].

Data from the Swedish Registry of Births reveal that premature babies with gestational age between 22 and 27 weeks have an uncomplicated survival rate at 18–43 years of 22.3% compared to 63% in full-term newborns [13,16]. Infants born extremely preterm have been shown to experience worse socioeconomic outcomes than those born at term.

The mode of birth can have an impact on the incidence of complications. Cesarean delivery is an important risk factor for the mother, such as intrapartum hemorrhages or injuries, but also a risk for subsequent pregnancies, such as the need for repeated cesarean section, placenta accreta, or placenta praevia. Based on the results of a large study on births between 22 and 26 weeks, cesarean section delivery is recommended in this gestational age category only in cases with maternal or obstetric indication, such as abnormal presentation, HELLP syndrome, severe IUGR, fetal distress [9].

In Bell’s study, although the majority of infants were born by cesarean delivery (64.8%), marginally smaller proportions of births at 22 weeks (3.1%) and 23 weeks (39.7%) used this delivery method [2].

The aim of the present study was to assess mortality, in-hospital morbidities, and short-term outcome of preterm newborns at 22–25 weeks of gestation, born in 2021–2024, admitted in a level 3 unit, with developing care experience in this category of preterm newborns, and an annual increase in the number of patients in this category.

## 2. Methods

Prospectively collected maternal and neonatal data, spanning from birth to discharge, transfer, or death, were analyzed for the present study. Follow-up data were collected until 12 months corrected age.

This study enrolled 49 pre-term infants, born between 22 weeks 0 days and 25 weeks and 6 days, weighing 400 to 1000 g, admitted to the NICU of a tertiary-level unit, between 2021 and 2024.

The inclusion criteria were the gestational age ≤ 25 weeks of gestation, born in the unit, or transferred from another maternity ward. Preterm newborns with severe malformations that compromised the outcome and newborns that did not respond to neonatal resuscitation maneuvers at birth were not enrolled in the study.

### 2.1. Clinical Course and Interventions

Maternal pathologies, pregnancy, and delivery information were collected: type of pregnancy (single or multiple, spontaneous or in vitro fertilization), hypertension or preeclampsia, urinary infection, positive cultures, chorioamnionitis, premature rupture of membranes, antenatal corticosteroids, as well as information on mode of delivery, place of birth: inborn or outborn transferred after birth to the level 3 unit, all factors that can have impact on the outcome of extremely preterm infants.

All infants had their clinical interventions and morbidities while hospitalized recorded. We assessed the incidence of six main complications of preterm birth: respiratory distress syndrome (RDS), persistent ductus arteriosus (PDA), intraventricular hemorrhage (IVH), necrotizing enterocolitis (NEC), retinopathy of prematurity (ROP), and bronchopulmonary dysplasia (BPD). We also analyzed the incidence of early neonatal sepsis, digestive and pulmonary hemorrhages, as possible complications with an impact on the infant’s immediate and long-term outcome. For PDA, according to the unit’s protocol, all newborns were evaluated by ultrasound in the first 72 h.

The IVH severity was defined according to the Papile criteria [17], while for necrotizing enterocolitis (NEC), Bell’s classification stage was used [18], and for bronchopulmonary dysplasia (BPD), Jensen et al.’s definition was employed [19]. Jensen’s BDP definition uses the neonates state at 36 weeks’ postmenstrual age or at discharge home, if earlier: no BDP when no support or breathing room air; grade 1 when a nasal cannula at a flow rate of 2 L/min or lower is needed; grade 2 when nasal cannula at a flow rate above 2 L/min or noninvasive positive airway pressure; and grade 3 for invasive mechanical ventilation.

Retinopathy of prematurity (ROP) severity was quantified according to the criteria of the national guideline for ROP, and treatment with laser coagulation and/or intraocular vascular endothelial growth factor (VEGF) inhibitor was assigned. The death of an infant before hospital discharge was defined as neonatal mortality.

Active treatment of the study group was considered the administration of surfactant with follow-up of the number of doses, the therapy for the patent ductus arteriosus, the need for inotropic support on the first day of life, therapy of retinopathy of prematurity, and the need for blood transfusion and its possible correlation with the incidence of NEC or ROP. Surfactant therapy was administered according to the national and European guidelines for respiratory distress. As for PDA therapy, it was initiated after cardiac ultrasound, and the type of treatment administered varied depending on the possible contradictions at the time of initiation of treatment, but also on the response to the initial treatment.

### 2.2. Laboratory Data

For the study of the outcome of the enrolled patients, we analyzed the inflammatory markers commonly determined in premature infants hospitalized in our service. We analyzed the value of C-reactive protein (CRP) and procalcitonin (PCT) on the first and second day of life, which are routinely performed in preterm infants admitted to our NICU to assess neonatal inflammation in the context of maternal inflammation or infection. For CRP determination, we used the immunoturbidimetry method with a clinical chemistry analyzer (Berckman Coulter, AU 680, Chaska, MN, USA). For the PCT value, the chemiluminescence technique was used and applied with DXI (Beckman Coulter DXI, AU 680, Chaska, MN, USA).

### 2.3. Follow-Up

The premature infants were enrolled in the follow-up program after discharge from the maternity ward. The neurological development of each participant was assessed at 3 months, 6 months, and 12 months (corrected age) by a pediatric neurologist.

### 2.4. Statistical Analysis

Microsoft Excel was used to collect and organize the data. Statistical analyses were performed using IBM SPSS 25.0. Two-tailed tests were exclusively used, and a *p*-value < 0.05 was considered statistically significant.

Continuous variables were tested for normality using the Shapiro–Wilk test. The normally distributed variables are reported as mean ± standard deviation (SD), while non-normally distributed variables are presented as median and interquartile range (IQR). Categorical variables are reported as frequencies and percentages.

Student’s *t*-test or the Mann–Whitney U test, as appropriate, were used for group comparisons. Associations between categorical variables were evaluated through the χ^2^ test or, when expected cell counts were less than five, Fisher’s exact test.

To explore potential factors associated with mortality and major neonatal morbidities, exploratory multivariable logistic regression analyses were performed. Gestational age and birth weight were included as a priori adjustment variables, given their established clinical relevance in extremely preterm infants. Additional covariates were selected based on clinical plausibility and results from univariable analyses. Results are reported as odds ratios (OR) with 95% confidence intervals (95% CI).

A two-sided *p*-value < 0.05 was considered statistically significant.

Ethical approval was granted by the unit’s ethics committee, and written informed consent was obtained from the parents of all participating infants.

## 3. Results

### 3.1. Study Population Morbidities, Pregnancy, and Delivery Data

The study group consisted of 49 premature infants with a mean gestational age of 24.37 ± 0.76 weeks, and birth weight of 665 ± 143 g, Apgar score of 3 (2–5) at 1 min and 5 (4–6) at 5′, 26 (53.1%) male newborns. In the delivery room, most of the newborns were intubated (43/49), and six had only T-piece ventilation applied. Extended resuscitation was applied for only one patient (Table 1).

The deliveries were almost equally proportioned by cesarean section and vaginal deliveries (49% vs. 51%). Single pregnancies (89%) predominated, spontaneous were 41/49 (83.7%), and most of the cases were inborn births (81.9%). In 13 cases (26.5%), maternal corticoid was not given. In 26 (53.1%) cases, maternal chorioamnionitis was present, and premature rupture of membranes (>18 h) presented 20 cases (40.8%). 4 (8.2%) mothers had pre-eclampsia, and 2 (4.1%) mothers had a urinary infection before delivery (Table 2).

The role of prenatal care on the mortality of the group was assessed. No statistically significant association was observed between the type of pregnancy surveillance and neonatal mortality (*p* = 0.551). Although mortality was higher among infants born after incomplete or no prenatal care and pregnancy surveillance, likely due to the low number of fatal events, this difference was not statistically significant.

Most neonatal in-hospital morbidities were comparable across gestational age categories. All newborns had RDS, 85.7% severe form, 91.8% had PDA, and 26/45 with PDA were treated. At 13 (26.5%), newborns with NEC were present. The IVH was severe, third and fourth grade in 7 (14.3%) and 9 (18.4%) cases, respectively.

As far as chronic complications are concerned, BPD developed 67.3%, and ROP 57.1% of cases.

The mortality rate in the group was 28.5%, and of the 14 deaths, four died in the first 72 h, and two occurred late, at the age of 4 months. In the latter, the cause was severe BPD, with pulmonary hypertension (Table 3).

All preterm infants received one dose of surfactant in the first 6 h of life, and 33/49 received a second course at 12 h of age.

Treatment of persistent ductus arteriosus was applied to 26 cases. In seven cases, only paracetamol was administered, in five cases only ibuprofen, and in eight cases a combination of paracetamol and ibuprofen.

The correlation between PDA and its effect on BPD development was analyzed, and no statistically significant association was observed between the type of PDA treatment and BPD. However, a trend toward statistical significance was identified, with a higher proportion of BPD infants receiving combination therapy compared to those receiving monotherapy or no treatment (*p* = 0.094).

The NEC incidence in the study group was 26.5%. Were considered positive diagnosis for NEC all forms according to Bell’s criteria. No statistically significant associations were observed between NEC and maternal preeclampsia or chorioamnionitis (*p* > 0.05). The need for inotropic support in the first 24 h of life correlates with compromised hemodynamics that can facilitate de NEC. No association was observed between NEC and the need for inotropic support or mortality (*p* > 0.05).

Grade III and IV of IVH were present in seven (14.3%) and nine (18.4%) of the preterm infants in the study group, respectively. No statistically significant association was observed between intraventricular hemorrhage grade and mortality (*p* = 1.000). Although deaths occurred across multiple IVH grades, their distribution did not differ significantly between categories, most likely due to the low number of fatal events in the study cohort.

The oxygen requirement was analyzed at three timepoints: day 1, day 2, and at 28 days of life. Considering the impact of oxygen therapy on chronic complications, its influence on BPD and ROP at the three time points was assessed. FiO_2_ requirement on day 1 of life was not associated with BPD or retinopathy (*p* > 0.05). In contrast, higher FiO_2_ requirements at day 28 were significantly associated with BPD (*p* = 0.003), ROP (*p* = 0.003), and the need for ROP therapy (*p* = 0.039). The severity of RDS was not significantly associated with these outcomes (*p* > 0.05).

The enrolled patients required multiple blood transfusions during hospitalization for anemia treatment. The number of transfusions was significantly higher in infants with ROP compared to those without retinopathy (four [3,8,12] vs. two [4,20], *p* = 0.011). Similarly, infants who developed NEC required a significantly higher number of transfusions compared to those without NEC (six [2,21] vs. three [4,20], *p* = 0.004).

ROP treatment was performed by laser photocoagulation and/or intraocular VEGF inhibitor according to ROP treatment guidelines. Among infants with ROP, no statistically significant association was observed between laser therapy and treatment with Avastin (OR = 1.00, *p* = 1.000). The distribution of therapeutic approaches was similar, suggesting independent use based on clinical indications.

We assessed the eventual role of early complications like severe bleeding: pulmonary or digestive on the mortality rate. Pulmonary hemorrhage was significantly associated with increased mortality. (53.8% vs. 19.4%; OR = 4.83, *p* = 0.031).

Although mortality was higher among infants with digestive hemorrhage compared to those without this complication (37.5% vs. 24.2%), the association did not reach statistical significance (OR = 1.88, *p* = 0.501).

The need for inotropic support in the first 24 h did not influence the mortality rate of the study group. (*p* > 0.05).

In the adjusted model for mortality, pulmonary hemorrhage remained independently associated with death (adjusted OR 7.86, 95% CI 1.31–47.37, *p* = 0.024), while birth weight showed a borderline protective effect (*p* = 0.065). For major morbidities, the number of transfusions remained independently associated with both retinopathy of prematurity (adjusted OR 1.27, 95% CI 1.01–1.59, *p* = 0.045) and necrotizing enterocolitis (adjusted OR 1.36, 95% CI 1.09–1.69, *p* = 0.006).

Univariate and multivariable logistic regression analyses were performed for mortality, retinopathy of prematurity, and necrotizing enterocolitis. Multivariable models were adjusted for gestational age and birth weight, with additional covariates selected based on clinical relevance and univariable analysis. Birth weight was modeled per 100 g increase.

Univariate and exploratory multivariable logistic regression analyses were performed to evaluate factors associated with mortality and major neonatal morbidities. In the adjusted model for mortality, pulmonary hemorrhage remained independently associated with death (adjusted OR 7.86, 95% CI 1.31–47.37, *p* = 0.024), while birth weight showed a borderline protective effect (*p* = 0.065). For major morbidities, the number of transfusions remained independently associated with both retinopathy of prematurity (adjusted OR 1.27, 95% CI 1.01–1.59, *p* = 0.045) and necrotizing enterocolitis (adjusted OR 1.36, 95% CI 1.09–1.69, *p* = 0.006).

Inflammation in premature births is a common process. We analyzed the value of the C-reactive protein (CRP) and procalcitonin (PCT) inflammatory markers on the first and second day of life and their impact on different morbidities in the patients of the group (Table 4).

Procalcitonin levels on day 2 were not significantly associated with necrotizing enterocolitis (*p* = 0.430). However, significantly lower PCT-DOL 2 values were observed among infants who developed bronchopulmonary dysplasia (*p* = 0.013) and retinopathy of prematurity (*p* = 0.039). Although higher PCT-DOL2 levels were observed among non-survivors compared to survivors, this difference did not reach statistical significance (*p* = 0.104).

### 3.2. Follow-Up Early Outcome

The infants were enrolled in a follow-up program after discharge. The follow-up was performed at 3 different time points: at 3 months, 6 months, and 12 months (corrected age). The follow-up data are shown in Table 5.

The rate of participants in the program was 51% at the first time point. At this first evaluation, 15 (60% of participants at follow-up) patients had normal neurological development. At 12 months of age, however, the neurological examination was normal in only three patients (23.08%), but a much smaller number of patients were presented, only 36.5% of the study group. Although participation in this evaluation was significantly lower than in the first evaluation, neurodevelopmental disorders were present in 10 of the patients, and one had a severe form of neurodevelopmental delay with spastic diplegia. The findings reported need to be interpreted with considerable caution, given the high rate of attrition at follow-up.

## 4. Discussion

The study allowed the analysis of the morbidity and evolution in the first year of life of a category of premature infants who are growing in our geographical region.

The incidence of premature infants with gestational age ≤ 25 weeks showed an increasing trend during the study, from four cases in the first year to 25 cases in the last year (2024) of the study.

Among preterm infants ≤ 25 weeks of gestation born in 2021–2024 and treated in our 3rd-level unit, 71.4% survived to hospital discharge. Five infants of the group died in the first 72 h after birth.

The survival rate in the study group of 71.4% is close to that reported in Bell et al.’s study of 78.3% [2]. We must consider 2 aspects related to this study: the analysis was on a much larger group of patients, and the gestational age of the patients included in the study was more extended, including infants between 22 and 26 weeks being enrolled. In terms of mortality, they analyzed the number of deaths in the first 12 h, respectively, until discharge. In our study, only one patient (1/49) died within the first 12 h after birth, while in those with 25-week GA the survival rate was 84%. According to the British Association for Perinatal Medicine (BAPM) framework for practice, overall perinatal survival among infants admitted to the NCU is approximately 45% at 23 weeks of gestation, 63% at 24, 77% at 25, and 84% at 26 [16,22,23].

In Sasaki et al.’s study from Japan, the mortality rate during hospitalization of the group was 14%, but patients with gestational age up to 27 + 6 D were enrolled, i.e., including gestational ages 2 weeks higher than our group, with a GA average of 25.2 weeks compared to 24.37 in the current study [24].

All infants had RDS and received at least one dose of surfactant. PDA present in the important part of the lot, even if it was hemodynamically significant, had no influence on the long-term evolution, although those who received multiple medication for PDA had a higher frequency of BPD. Medication for PDA closure in our study was higher (53%) than in Bell’s study [2], where treatment was given to 23.4%. In their study, except for medication, surgical closure was done in 7.4% of patients. Correlation of BPD with the significant PDA early life of preterm infants, being well known, was explored in the current study [23,25]. Infants with PDA requiring treatment developed BPD more often than those with untreated PDA (73% vs. 63.1%).

BPD was present at 67.3% of the group, with decreasing incidence with increasing gestational age, like data from other studies. The association between the number of blood transfusions and the incidence of retinopathy is of interest. Although the number of ROP cases was lower at the group level (57.1%), compared to other studies that at similar gestational age report much higher incidences, of over 90% [2,26], more than half of patients with ROP required laser therapy or administration of VEGF inhibitor [27,28].

The present cohort showed a higher incidence of necrotizing enterocolitis than the 2008–2012 cohort reported by Stoll et al. [5]. We must consider that in our study, we enrolled only patients with gestational age ≤ 25, and enrolled the mild form of NEC’s as well [8]. The link between inflammation at birth and NEC’s incidence was found in this study, like other studies, where increased inflammatory markers at birth, maternal chorioamnionitis, were proven to be risk factors for NEC [29,30].

Survivors in the study group adhered to the follow-up program in a proportion of 51%, a percentage lower than the data of other authors (62.9%) [4,21,27].

The most severe neurological complication encountered in the study group was spastic diplegia. However, it should be noted that substantial loss to follow-up at 12 months means that the observed distribution of outcomes, with 23.08% of assessed participants showing normal development and the majority presenting only developmental coordination disorders, cannot be taken as representative. The absence of standardized neurodevelopmental assessment at 24 months using age-specific developmental scores further limits the conclusions that can be drawn from this study. These neurodevelopmental data should therefore be regarded as exploratory and not definitive.

Comparing neurodevelopmental results with other studies is difficult, owing to methodological differences such as variable inclusion criteria (gestational age, birth years, and other factors) or the assessment instruments and their versions. Sociodemographic factors also differ between studies, as does the population’s adherence to the follow-up programs, which vary between countries. Finally, the definitions of neurodevelopmental impairment (NDI) used also vary by study [23]. Such factors may result in substantial differences in reported rates of NDI and severe NDI [10,16].

For example, the EPICure 2 study followed children born in the United Kingdom at below 26 weeks’ gestational age through to age 2.5–3 years, reporting a severe NDI rate of 19%; however, this figure was based on a definition limited to developmental quotients more than 3 SDs below the mean [4,5]. In another study, the Neonatal Research Network of Japan reported outcomes for children born between 2008 and 2012 at below 25 weeks’ gestational age, documenting an NDI rate of 38.1% among those who completed a neurodevelopmental follow-up assessed via a developmental test specifically standardized for Japanese children. The follow-up rate reported in the Japanese study was 60.6% [1].

In the present study, neurodevelopmental follow-up was conducted solely at 12 months of age, with a lower participation than that observed in the Japanese study. The considerably higher rate of neurodevelopmental impairment reported here should therefore be interpreted with caution, as the reduced participation in the follow-up likely inflated the apparent NDI rate. Direct comparison with the Japanese cohort and other large international studies is further limited by the small, local, and exploratory nature of the present sample, precluding any inference of equivalence from larger and more robustly followed cohorts.

In the Epipage 2 study conducted in France, premature infants with gestational ages of 24 and 25 weeks evaluated at the age of 5 years, presented severe NDI with cerebral palsy in 25% and 15%, respectively, and over 30% and 20%, respectively, associated with other severe disabilities [7]. Proportions of children with developmental coordination disorders were 18.8% in the 24–26 weeks of gestational age. In the Eipage 2 study, preterm infants below 24 weeks of gestation were not included.

The Swedish EXPRESS study, which followed children born in 2004–2007 at below 27 weeks’ gestational age, found that 58% exhibited some degree of disability at 30 months’ corrected age. Severe disability was identified in only 11% of cases; however, the definition used included Bayley-III composite cognitive, language, or motor scores falling more than 3 SDs below the mean [28].

Motor development following extreme preterm birth can be disrupted across a wide spectrum, ranging from minor delays in milestones such as sitting or walking to severe neuromotor impairment. Cerebral palsy, the most significant motor disorder, affects around 10–20% of preterm individuals. In our study, motor development problems were identified in 20% of patients; however, given the follow-up rate of 51% at the initial assessment, decreasing to 26.5% at 12 months, this figure is subject to attrition bias and should not be interpreted as an estimate of the true prevalence within the cohort.

The principal strength of this study lies in its prospective characterization of morbidity and hospital outcomes in a cohort of infants born at or below 25 weeks of gestation within a single level 3 center. The inclusion of neurodevelopmental follow-up at 12 months, whilst limited by significant attrition, provides preliminary exploratory data that may inform the design of future studies with more complete follow-up and standardized assessment beyond 12 months of age.

## 5. Limitations

The study was conducted in a single center.

No formal a priori sample size calculation was performed, as the study was designed as a prospective observational cohort including all eligible extremely preterm infants admitted consecutively during the study period. Consequently, statistical power may have been insufficient to detect associations for some outcomes with a low event rate. Although exploratory multivariable analyses were performed to adjust for major confounders such as gestational age and birth weight, the relatively small number of outcome events limited the number of predictors that could be included in the models. Therefore, the multivariable analyses should be interpreted with caution and regarded primarily as hypothesis-generating.

The 12-month-end points presented provide a relatively limited preview of the future. Future work should aim to extend the follow-up to later ages, improve participant retention, and incorporate standardized neurocognitive evaluation tools appropriate for older children, which will allow us to obtain more robust and accurate data related to the NDI of the study group

## 6. Conclusions

Among preterm infants ≤ 25 weeks of gestation born in 2021–2024 treated in our unit, the survival rate at discharge was like other larger studies.

The low follow-up rate in the study group does not allow us to generalize the conclusions, but based on current data, the incidence of NDI was like other studies.

Infants born extremely preterm continue to pose a disproportionate challenge in terms of adverse outcomes and resource utilization, with considerable scope for improvement across both diagnostic and care. The inherently small numbers of extremely preterm births within any single center or region make the conduct of targeted randomized controlled trials particularly challenging. As the gestational threshold at which active intervention is considered appropriate shifts progressively earlier, the demand for research into optimizing the management of the extremely preterm infant population is set to increase. Although advances in neonatal care and the consolidation of high-level NICU services have improved survival rates for some extremely preterm infants, the risk of life-long, significant morbidity remains disproportionately high for this patient group.

## Figures and Tables

**Table 1:** Demographics and delivery room approach of infants.

Variable	22 w (n = 1)	23 w (n = 5)	24 w (n = 18)	25 w (n = 25)	Total (n = 49)	*p*-Value
Sex						0.582
Male	1 (100)	3 (60.0)	9 (50.0)	13 (52.0)	26 (53.1)	
Female	0	2 (40.0)	9 (50.0)	12 (48.0)	23 (46.9)	
Birth weight (g)	490 (490–490)	490 (490–500)	635 (590–690)	740 (680–840)	680 (550–740)	0.001
Apgar score at 1 min	2 (2–2)	3 (2–4)	3 (2–5)	3 (2–5)	3 (2–5)	0.81
Apgar score at 5 min	4 (4–4)	5 (4–6)	5 (4–6)	5 (4–6)	5 (4–6)	0.77
Delivery room resuscitation						0.655
Neopuff	0	0	2 (11.1)	4 (16.0)	6 (12.2)	
ET	1 (100)	5 (100)	15 (83.3)	21 (84.0)	42 (85.7)	
ET + medication	0	0	1 (5.6)	0	1 (2.0)	
Place of birth (Inborn)	1 (100)	5 (100)	15 (83.3)	19 (76.0)	40 (81.6)	0.591

w = weeks of gestation; ET = endotracheal intubation.

**Table 2:** Maternal, pregnancy, and delivery characteristics of the study population (n = 49).

Characteristics of Pregnancy and Delivery, No, Total (%)	22 w (n = 1)	23 w (n = 5)	24 w (n = 18)	25 w (n = 25)	Total (n = 49)	*p*-Value
Delivery mode						0.385
Vaginal	1 (100)	4 (80.0)	8 (44.4)	12 (48.0)	25 (51.0)	
Cesarean section	0	1 (20.0)	10 (55.6)	13 (52.0)	24 (49.0)	
Pregnancy type						0.568
Singleton	1 (100)	5 (100)	17 (94.4)	21 (84.0)	44 (89.8)	
Multiple	0	0	1 (5.6)	4 (16.0)	5 (10.2)	
Survey of pregnancy						0.844
Complete	1 (100)	4 (80.0)	11 (61.1)	14 (56.0)	31 (63.3)	
Incomplete	0	1 (20.0)	4 (22.2)	8 (32.0)	13 (26.5)	
None	0	0	3 (16.7)	2 (8.0)	5 (10.2)	
Preeclampsia	0	0	3 (16.7)	1 (4.0)	4 (8.2)	0.416
Chorioamnionitis	1 (100)	4 (80.0)	8 (44.4)	13 (52.0)	26 (53.1)	0.409
Urinary infection	0	1 (20.0)	1 (5.6)	0	2 (4.1)	0.217
IVF pregnancy	1 (100)	1 (20.0)	1 (5.6)	5 (20.0)	8 (16.3)	0.074
Corticoid						0.388
Complete	1 (100)	4 (80.0)	7 (38.9)	15 (60.0)	27 (55.1)	
Incomplete	0	1 (20.0)	3 (16.7)	5 (20.0)	9 (18.4)	
None	0	0	8 (44.4)	5 (20.0)	13 (26.5)	
PROM (Yes)	1 (100)	4 (80.0)	4 (22.2)	11 (44.0)	20 (40.8)	0.21

w = weeks of gestation; PROM—premature rupture of membranes, IVF—in vitro fertilization.

**Table 3:** In hospital morbidity, mortality, and interventions of the study group.

Outcome	22 w (n = 1)	23 w (n = 5)	24 w (n = 18)	25 w (n = 25)	Total (ng = 49)	*p*-Value
RDS severe	1 (100)	5 (100)	14 (77.8)	22 (88.0)	42 (85.7)	0.566
Mechanical ventilation (hours)	720 (720–720)	500 (300–720)	250 (120–480)	120 (48–240)	180 (72–360)	0.353
FiO_2_ DOL1	100 (100–100)	80 (60–100)	60 (40–80)	40 (30–60)	50 (40–80)	0.325
FiO_2_ DOL2	100 (100–100)	70 (50–90)	50 (40–70)	35 (25–50)	45 (30–70)	0.417
FiO_2_ DOL28		30 (25–40)	25 (21–35)	21 (21–30)	25 (21–35)	0.11
PDA	1 (100)	5 (100)	16 (88.9)	23 (92.0)	45 (91.8)	0.756
PDA treatment	1 (100)	4 (80.0)	12 (66.7)	16 (64.0)	33 (67.3)	0.114
NEC	0	1 (20.0)	4 (22.2)	8 (32.0)	13 (26.5)	0.077
IVH						
grade I	1 (100)	1 (20.0)	8 (44.4)	16 (64.0)	26 (53.1)	0.139
grade II	0	3 (60.0)	2 (11.1)	2 (8.0)	7 (14.3)	
grade III	0	1 (20.0)	4 (22.2)	2 (8.0)	7 (14.3)	
grade IV	0	0	4 (22.2)	5 (20.0)	9 (18.4)	
Inotropic support	1 (100)	4 (80.0)	15 (83.3)	24 (96.0)	44 (89.8)	0.568
Pulmonary hemorrhage	1 (100)	1 (20.0)	4 (22.2)	7 (28.0)	13 (26.5)	0.795
Digestive hemorrhage	1 (100)	2 (40.0)	4 (22.2)	9 (36.0)	16 (32.7)	0.647
BPD	0	1 (20.0)	8 (44.4)	24 (96.0)	33 (67.3)	0.76
ROP	1 (100)	4 (80.0)	9 (50.0)	14 (56.0)	28 (57.1)	0.531
ROP requiring laser	0	0	4 (22.2)	7 (28.0)	11 (22.4)	0.56
Avastin treatment	0	0	1 (5.6)	1 (4.0)	2 (4.1)	0.042
Death	1 (100)	2 (40.0)	5 (27.8)	6 (24.0)	14 (28.6)	0.077

RDS = respiratory distress syndrome; FiO_2_ DOL = oxygen concentration on day of life 1, 2, 28; PDA = persistent ductus arteriosus; NEC = necrotizing enterocolitis; IVH = intraventricular hemorrhage, BPD = bronchopulmonary dysplasia; ROP = retinopathy of prematurity.

**Table 4:** Inflammation biomarkers in the study group.

Variable	n	Descriptive Statistics
CRP DOL1 (mg/dL)	49	0.03 (0.02–0.10)
PCT DOL1 (ng/mL)	49	0.77 (0.43–2.50)
CRP DOL2 (mg/dL)	48	0.30 (0.08–1.44)
PCT DOL2 (ng/mL)	48	23.18 (4.92–72.39)

CRP DOL1&2 = C-reactive protein day of life 1&2, PCT DOL1&2 = Procalcitonin day of life 1&2. (Second-day data are unavailable for one patient who died on day one of life).

**Table 5:** Neurodevelopmental outcome of the infants.

	Follow-Up 1 3 M (n = 25)	Follow-Up 2 6 M (n = 21)	Follow-Up 3 9 M (n = 13)
Normal	15 (60)	10 (47.62)	3 (23.08)
Developmental coordination disorder	5 (20)	10 (47.62)	9 (69.23)
Hypotonia	3 (12)	-	-
Spastic diplegia/diparesis	2 (8)	1 (4.76)	1 (7.69)

M = months.

## Data Availability

The original contributions presented in this study are included in the article. Further inquiries can be directed to the corresponding author.
